# Learning Guided Binary PSO Algorithm for Feature Selection and Reconstruction of Ultrasound Contrast Images in Endometrial Region Detection

**DOI:** 10.3390/biomimetics10090567

**Published:** 2025-08-25

**Authors:** Zihao Zhang, Yongjun Liu, Haitong Zhao, Yu Zhou, Yifei Xu, Zhengyu Li

**Affiliations:** 1 School of Computer Science and Engineering, Suzhou University of Technology, Suzhou 215500, China; aebestach@stu.sjzu.edu.cn (Z.Z.); zyntcs2022@163.com (Y.Z.); 15501325489@163.com (Y.X.); 2 School of Computer Science and Engineering, Shenyang Jianzhu University, Shenyang 110168, China; lizhengyu@sjzu.edu.cn

**Keywords:** swarm intelligence, BPSO, feature-level fusion, endometrial lesions, YOLO

## Abstract

Accurate identification of the endometrial region is critical for the early detection of endometrial lesions. However, current detection models still face two major challenges when processing endometrial imaging data: (1) In complex and noisy environments, recognition accuracy remains limited, partly due to the insufficient exploitation of color information within the images; (2) Traditional Two-dimensional PCA-based (2DPCA-based) feature selection methods have limited capacity to capture and represent key characteristics of the endometrial region. To address these challenges, this paper proposes a novel algorithm named Feature-Level Image Fusion and Improved Swarm Intelligence Optimization Algorithm (FLFSI), which integrates a learning guided binary particle swarm optimization (BPSO) strategy with an image feature selection and reconstruction framework to enhance the detection of endometrial regions in clinical ultrasound images. Specifically, FLFSI contributes to improving feature selection accuracy and image reconstruction quality, thereby enhancing the overall performance of region recognition tasks. First, we enhance endometrial image representation by incorporating feature engineering techniques that combine structural and color information, thereby improving reconstruction quality and emphasizing critical regional features. Second, the BPSO algorithm is introduced into the feature selection stage, improving the accuracy of feature selection and its global search ability while effectively reducing the impact of redundant features. Furthermore, we refined the BPSO design to accelerate convergence and enhance optimization efficiency during the selection process. The proposed FLFSI algorithm can be integrated into mainstream detection models such as YOLO11 and YOLOv12. When applied to YOLO11, FLFSI achieves 96.6% Box mAP and 87.8% Mask mAP. With YOLOv12, it further improves the Mask mAP to 88.8%, demonstrating excellent cross-model adaptability and robust detection performance. Extensive experimental results validate the effectiveness and broad applicability of FLFSI in enhancing endometrial region detection for clinical ultrasound image analysis.

## 1. Introduction

Endometrial lesions are among the most common gynecological diseases and pose a significant diagnostic and therapeutic challenge due to the heterogeneity of their clinical manifestations. Conventional diagnostic methods primarily rely on manual imaging and manual histopathological analysis, which are time-consuming, labor-intensive, and sometimes suboptimal in clinical decision-making.

Among various imaging modalities, magnetic resonance imaging (MRI) is commonly used to diagnose endometrial lesions, but its limitations in terms of cost and examination time make it less accessible. Transvaginal sonography (TVS), by contrast, is a preferred technique due to its simplicity, cost-effectiveness, and non-invasive nature. Contrast-enhanced ultrasound (CEUS), as a pure blood pool imaging technique, effectively reveals perfusion differences between lesions and surrounding tissues, offering substantial clinical value [1,2]. However, deep learning research based on transvaginal CEUS imaging remains limited, with most studies still focusing on MRI, CT, TVS, or multimodal fusion methods.

Current research on endometrial region detection based on transvaginal contrast-enhanced ultrasound imaging remains limited. To enrich the solutions space for this problem, we propose a feature-level image fusion approach combined with a swarm intelligence optimization algorithm to reconstruct images for training YOLO-based deep learning models.

The motivation for incorporating feature-level fusion stems from the growing body of evidence supporting the importance of feature engineering in medical image analysis. Prior studies have demonstrated that carefully designed feature representations can significantly enhance diagnostic accuracy. For instance, Zhang et al. [3] proposed a multi-stage framework that first computes novel features and then maps them into input images for a ResNet50 neural network. This approach achieved 100% classification accuracy on both the Coimbra and Wisconsin datasets, demonstrating the potential of feature engineering in improving diagnostic performance. In parallel, Essa et al. [4] have explored machine learning techniques for feature extraction and selection during feature engineering, as well as methods for addressing imbalanced datasets and integrating multi-modal data in statistical modeling. These studies emphasize that the use of robust machine learning strategies to optimize feature selection, manage data imbalance, and enhance the stability and interpretability of models is crucial for advancing radiomics. Collectively, these findings underscore the pivotal role of feature engineering in driving progress toward more accurate and non-invasive diagnostic tools, and suggest that an ideal feature selection method should not only effectively capture endometrial feature information, but also ensure robustness, interpretability, and generalizability of the imaging process across diverse clinical scenarios.

In recent years, Convolutional Neural Networks (CNNs), a cornerstone of deep learning, have demonstrated remarkable success in medical image analysis, particularly in tasks such as segmentation, classification, and lesion detection. Deep learning methods also play a significant role in the early detection of endometrial lesions. Mao et al. [5] developed a deep learning-based automatic segmentation model using U-Net and conducted experiments on magnetic resonance imaging (MRI) data from 117 patients with endometrial carcinoma (EC), proposing an effective early EC staging method. Coada et al. [6] utilized preoperative CT images of endometrial cancer patients for radiomic feature extraction and employed three machine learning models to predict the disease-free survival (DFS) of patients. Yang et al. [7] applied various classical machine learning and deep learning algorithms to classify and predict pathological tissue images of low-grade endometrial stromal sarcoma (LGESS). Chen et al. [8] used YOLOv3 to detect lesion areas in MR images of EC patients, followed by ResNet to evaluate the depth of invasion.

The YOLO family, as an efficient end-to-end object detection framework, shows strong potential in medical image segmentation. YOLO11 enhances YOLOv8 with modules like SPPF, C2PSA, and C3k2 to improve attention, training stability, and speed. YOLOv12 further advances performance with Area Attention, R-ELAN, FlashAttention, and large-kernel convolutions, boosting global modeling and real-time capability. YOLO11 and YOLOv12 offer greater accuracy and efficiency for complex medical imaging tasks.

However, when applying YOLO and feature engineering to contrast-enhanced ultrasound (CEUS) images, several unexpected issues emerged. Specifically, the model frequently misidentified surrounding tissues as endometrial regions, leading to over-detection and a relatively low overall detection accuracy. Further analysis revealed that these limitations were primarily due to two factors: the insufficient exploitation of the unique color and texture characteristics inherent in CEUS images, and the use of suboptimal feature selection strategies. To address the latter, dimensionality reduction techniques such as Principal Component Analysis (PCA) can be leveraged to eliminate redundant and noisy features, thereby enhancing the discriminative capacity of the selected features. However, PCA alone may not fully capture the complex and nonlinear relationships in CEUS data, necessitating more advanced optimization strategies for robust feature selection.

Swarm Intelligence Optimization Algorithm (SI) is a central theory in the field of artificial intelligence, first proposed in 1989 by scientists Gerardo Beni and Jing Wang [9] initially applied to the study of cellular robotic systems. In nature, many biological groups such as ant colonies, schools of fish and flocks of birds, even if their individual abilities are limited, show remarkable problem-solving abilities when they join together to form collectives. This collective cooperation is evident not only in survival activities such as hunting and defense, but also in overcoming challenges that exceed the cognitive capabilities of individual members.

SI play a crucial role in feature selection (FS), especially when facing high-dimensional data. Traditional exhaustive or greedy methods are often inefficient for selecting optimal feature subsets. Recent research [10] has shown that a hybrid algorithm combining the salp swarm algorithm (SSA) and grasshopper optimization algorithm (GOA), known as crossover-salp swarm with grasshopper optimization (cSG), achieves superior performance. By introducing crossover operators to enhance population diversity and using SSA to guide local search, cSG effectively avoids premature convergence and local minima. Experimental results across benchmark datasets and real-world problems confirm the effectiveness and flexibility of the proposed method.

Among swarm-based optimization techniques, Particle Swarm Optimization (PSO) has been widely recognized for its simplicity, strong global search capability, and fast convergence. Inspired by the social behavior of bird flocking and fish schooling, PSO simulates a population of candidate solutions (particles) that explore the search space by updating their velocities and positions based on both individual and collective experiences. This balance between exploration and exploitation makes PSO particularly suitable for complex optimization tasks such as feature selection, where it can efficiently search large solution spaces for informative and compact feature subsets.

Building upon this foundation, the Binary Particle Swarm Optimization (BPSO)-based [11] medical image feature fusion method proposed in this study targets two key aspects of feature-level fusion for endometrial features in transvaginal contrast-enhanced ultrasound (CEUS) images. First, it enhances the representativeness of endometrial features by optimizing the selection of feature vectors. Second, the fused feature vectors are used to reconstruct images, which are subsequently employed in the final training stage of the YOLO-based target detection model.

The contribution of the research lies in

(1) Extending the Two-dimensional PCA(2DPCA) [12] algorithm to RGB three-channel, structural and color-fused image features are extracted from the three color channels of both conventional and contrast-enhanced ultrasound images to enhance the characterization of endometrial regions.
(2) During the feature selection stage, the traditional Binary Particle Swarm Optimization (BPSO) algorithm is incorporated with RGB images and 2DPCA features to screen projection vectors, thereby reconstructing images while preserving critical feature information. An enhanced BPSO strategy is developed to mitigate slow convergence and local optima trapping via optimized parameter updating, improving feature selection efficiency and robustness.
(3) The proposed FLFSI algorithm effectively enhances feature reconstruction quality and downstream recognition accuracy, demonstrating seamless compatibility with mainstream detection models while exhibiting superior cross-model adaptability and detection performance.

The remainder of the paper follows this format: Section 2 introduces related work, covering 2DPCA, feature selection, and SI in medical applications and their limitations. Section 3 elaborates on the methodology, detailing problem modeling, the FLFSI algorithm, and time-complexity analysis. Section 4 presents experiments, parameter discussions, and comparative results. Section 5 ends the study by explaining future work.

## 2. Related Work

### 2.1. Deep Learning Applications and Limitations in Endometrial Medical Imaging

As described in the introduction, most deep learning studies in the endometrial field are based on magnetic resonance imaging, endometrial histopathological sections, and traditional ultrasound. For instance, Takahashi et al. [13] developed an artificial intelligence-based system utilizing deep neural networks to automatically detect endometrial cancer regions in hysteroscopic images from 177 patients with various gynecological diseases. They proposed a continuity-analysis method to improve diagnostic accuracy and explored the combination of multiple network models. Results demonstrated an increase in accuracy from approximately 80% (standard method) to 89% (continuity analysis) and over 90% (combined models), while achieving sensitivities and specificities of 91.66% and 89.36%, respectively. Meanwhile, Sun et al. [14] developed HIENet, a computer-aided diagnosis (CAD) method based on a convolutional neural network (CNN) and attention mechanisms for the analysis of endometrial cancer histopathological images. In internal and external validations, HIENet achieved accuracies of 76.91% ± 1.17% and 84.50% for the four-class classification of endometrial tissues, and demonstrated high specificities (94.78% ± 0.87% and 100%) and good sensitivities (81.04% ± 3.87% and 77.97%) in the binary classification task for detecting endometrioid adenocarcinoma. This method outperformed three human experts and five CNN-based classifiers in overall classification performance and provided better diagnostic interpretability by highlighting the correlation between local pixel-level image features and morphological characteristics.

By reviewing relevant literature, we found that there is currently a lack of research on applying deep learning to transvaginal ultrasound endometrial imaging. This paper aims to enrich the solution to this problem, taking advantage of the inherent advantages of CEUS in providing dynamic perfusion information, and applying deep learning to the complex CEUS features of the endometrium. Specifically, the YOLO baseline model is used for experiments.

### 2.2. Feature Selection Applications and Limitations in Endometrial Other Medical Imaging

Feature selection plays a crucial role in machine learning. For instance, Zielinski et al. [15] utilized data from 13,933 questionnaires to predict endometriosis using LightGBM and other machine learning algorithms, enhancing model performance through multiple feature selection methods. Their final model achieved an AUC of 0.85 on the training set and 0.82 on the testing set, identifying key predictive factors such as cesarean section and ovarian cysts. When applied to medical imaging tasks, feature selection can also enhance downstream performance. For example, Brar et al. [16] analyzed both shallow and deep features, integrating them via a mid-level fusion strategy. They employed a novel Extra Tree–Whale Optimization Feature Selector (ET-WOFS) to accurately identify optimal features from heterogeneous data. This approach demonstrated strong effectiveness in providing accurate and reliable diagnostic support for endometrial cancer.

In addition to conventional feature selection methods, dimensionality reduction techniques such as 2DPCA can also serve as effective tools for identifying and extracting salient features, particularly in image-based data.

2DPCA is a dimensionality reduction algorithm designed for image feature extraction. Unlike traditional PCA, 2DPCA operates directly on image matrices, avoiding the need to convert images into vectors, which helps preserve spatial information and reduces computational complexity. By constructing a covariance matrix based on the original image and solving for its eigenvectors, 2DPCA determines optimal projection directions for effective feature representation. This approach is known for its efficiency and strong performance in capturing discriminative features, making it well-suited for tasks such as image recognition, face recognition, and object classification in computer vision.

Despite the advantages of 2DPCA in reducing dimensionality while preserving spatial structure, the method is not without limitations, especially when applied to complex medical imaging tasks such as endometrial lesion detection. By default, 2DPCA extracts principal components based on the eigenvectors of the image covariance matrix, projecting original image matrices onto a lower-dimensional subspace. While this can effectively capture global variance and reduce redundancy, the standard 2DPCA framework often neglects finer-grained, domain-specific characteristics critical for accurate lesion localization and classification.

One drawback lies in 2DPCA’s limited sensitivity to color and texture information. In clinical ultrasound or histopathological images of the endometrium, discriminative features are often subtle and encoded not only in structural patterns but also in color gradients and textural nuances. The conventional grayscale or single-channel input assumptions of 2DPCA inherently restrict its ability to fully utilize the rich information present in multi-channel images. As a result, the extracted features may fail to represent the intricate boundary details or the heterogeneous appearance of lesion regions, ultimately impacting downstream detection performance.

Another limitation arises from the fixed number of projection axes selected during 2DPCA-based feature extraction. The algorithm typically retains a pre-defined number of principal components based on eigenvalue thresholds or cumulative variance ratios. However, such static selection may not generalize well across diverse image sets, particularly when the variance is unevenly distributed across features. This can lead to the inclusion of redundant or irrelevant features and, conversely, the omission of diagnostically valuable but low-variance components. Consequently, when these features are fed into classification or detection models, the result is often suboptimal accuracy and generalization.

### 2.3. SI Applications and Limitations in Endometrial Other Medical Imaging

Binary Particle Swarm Optimization (BPSO) is a discrete adaptation of the Particle Swarm Optimization (PSO) algorithm, specifically designed for binary or combinatorial optimization problems. Unlike standard PSO, BPSO transforms the continuous position of particles using a sigmoid function to generate probabilities, which are then used to determine binary values (0 or 1) in each dimension. This approach allows the algorithm to operate effectively in a discrete search space. BPSO is known for its simplicity and fast convergence, and has been widely applied in areas such as feature selection, task scheduling, and network optimization.

In the field of deep learning, swarm intelligent optimization algorithms based on the behavior of biological groups have also been widely applied.

Chen et al. [17] utilized a deep learning model to predict the total electron content (TEC) of the ionosphere, employing the Firefly Assisted Multi-strategy Beluga Whale Optimization (FAMBWO) algorithm to optimize and select the hyperparameters of the deep learning model. Meng et al. [18] proposed a scheduling technique based on deep Q-learning and SI for path planning in unmanned aerial vehicles (UAVs). Patel, B.N. et al. [19] integrated a bee swarm optimization algorithm with deep learning for the diagnosis of pneumonia in chest X-rays, achieving higher accuracy than human experts alone. Additionally, Nagarajan, B. et al. [20] applied the Modified Gorilla Troops Optimization (MGTO) algorithm to the classification of oral squamous cell carcinoma (OSCC), marking the first use of a SI as an intermediate layer in deep learning, significantly enhancing OSCC classification performance.

Awotwe et al. [21] investigated the application of deep learning and swarm intelligence-based optimization algorithms in breast cancer diagnosis, proposing several integrated methods to enhance image classification and segmentation accuracy. Their approach combining TwinCNN with the Binary Ebola Optimization Search Algorithm achieved classification accuracies of 97.7% and 91.3% on histology and mammography datasets, respectively. In addition, the use of PSO with the improved fuzzy clustering algorithm ScARKFCM and the integration of ACO with ResNet-101 further demonstrated the effectiveness of combining optimization techniques with medical image analysis.

While BPSO demonstrates efficacy in discrete optimization problems, particularly for feature selection tasks by effectively identifying salient features within high-dimensional datasets, it exhibits significant computational inefficiencies in scenarios characterized by high feature dimensionality or large-scale data. This performance degradation is primarily attributed to the necessity of calculating and updating per-dimension value probabilities, resulting in computational complexity that scales sharply with increasing dimensionality.

## 3. Method

In the majority of medical imaging region detection tasks, predictive models are required to annotate the region of interest (ROI) within the image. Therefore, it is necessary to replace the detection head in the YOLO model’s Head framework with a segmentation head. Consequently, the YOLO11-Seg model was adopted as the baseline model for conducting experiments.

### 3.1. Problem Definition

**Figure 1:**
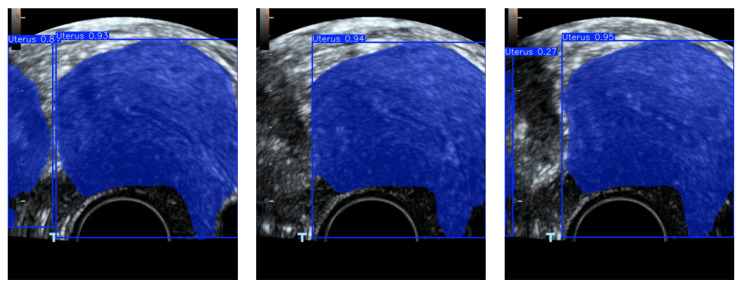
Error area detection case.

Accurate identification of the endometrial region in clinical ultrasound images is essential for the early diagnosis of endometrial lesions. However, as illustrated in Figure 1, this task faces several key challenges:

- Significant noise interference: Endometrial regions are often complex and exhibit blurred boundaries. Coupled with the presence of strong noise in ultrasound images, conventional detection models struggle to perform reliably under complex backgrounds.
- Insufficient feature representation: Traditional feature extraction methods, such as two-dimensional principal component analysis (2DPCA), are limited in their ability to capture comprehensive discriminative characteristics, particularly when integrating both color and structural information.
- High-dimensional redundancy: High-dimensional features may contain redundant or irrelevant information, which can negatively impact detection accuracy and reduce model training efficiency.

To address these challenges, we formalize the detection of the endometrial region as a feature selection and region recognition problem. Let the original image dataset be denoted as Equation (1):

(1) $$I=\left\{I_{1},I_{2},\ldots ,I_{n}\right\},\;I_{i}\in R^{H\times W\times C}$$

where *H*, *W*, and *C* represent the height, width, and number of channels (e.g., RGB) of each image.

For each image $I_{i}$, a feature extraction function f(·) is applied to obtain a *d*-dimensional feature vector Equation (2):

(2) $$F_{i}=f\left(I_{i}\right)\in R^{d}$$

The objective is to select a discriminative subset of features from the original high-dimensional feature space for training and evaluating the region detection model. A binary selection vector $S=\left[s_{1},s_{2},\ldots ,s_{d}\right]\in {\left\{0,1\right\}}^{d}$ is introduced, where:

- $s_{j}=1$ indicates the *j*-th feature is selected;
- $s_{j}=0$ indicates the *j*-th feature is discarded.

The selected feature vector is then expressed as Equation (3):

(3) $$F_{i}^{s}=F_{i}⊙S$$

where ⊙ denotes element-wise multiplication.

Thus, the task is formally defined as: Given a set of ultrasound images, identify an optimal binary feature selection vector *S* that retains compact and informative features to maximize region recognition performance.

### 3.2. FLFSI

#### 3.2.1. Feature Extraction in RGB Color Channels

From the study of 2DPCA, it is recognized that the covariance matrix plays a significant role in the fields of statistics and machine learning. This matrix effectively quantifies the covariance among multiple random variables, providing a crucial basis for analyzing the correlations between variables. However, 2DPCA only processes grayscale images when extracting principal components.

Considering the important clinical diagnostic value of the color information of the contrast images, the 2DPCA method was extended to the RGB three-channel space to fully utilize the feature information of the color images. In ultrasound images, there are differences in the reflectance characteristics of different tissues in the RGB spectrum, and the characteristics of endometrial blood perfusion can be better captured by analyzing multiple channels together. Meanwhile, synchronized processing of dynamic enhancement modes with different phases of the contrast agent can be achieved by creating a three-channel covariance matrix.

To this end, in both conventional and contrast-enhanced imaging, the covariance matrix is utilized for each channel to solve the projection vector for the corresponding channel, as defined by Equation (4):

(4) $$P^{RGB}\left(X\right)={\left(W^{RGB}\right)}^{T}S_{t}W^{RGB}$$

where, $W^{RGB}$ represents the projection vector containing three channels, and $S_{t}$ is the covariance matrix of the three-dimensional image. The expansion is shown in Equation (5). Assuming there are *N* training samples, the *i*-th training image is represented as $O_{i}\left(i=1,2,\ldots ,N\right)$, and the mean image of all training images is represented as $\bar{O}$.

(5) $$\left\{\begin{array}{c} S_{t}^{R}=\frac{1}{N}\sum_{i=1}^{N}{\left(O_{i}^{R}-\bar{O^{R}}\right)}^{T}\left(O_{i}^{R}-\bar{O^{R}}\right)\\ S_{t}^{G}=\frac{1}{N}\sum_{i=1}^{N}{\left(O_{i}^{G}-\bar{O^{G}}\right)}^{T}\left(O_{i}^{G}-\bar{O^{G}}\right)\\ S_{t}^{B}=\frac{1}{N}\sum_{i=1}^{N}{\left(O_{i}^{B}-\bar{O^{B}}\right)}^{T}\left(O_{i}^{B}-\bar{O^{B}}\right) \end{array}\right.$$

Next, compute the eigenvalues and eigenvectors of the covariance matrix in Equation (4). The eigenvectors obtained from this computation form the projection axes, which are then used in a matrix operation with the original image to derive the projected feature vector $Y^{RGB}$, as expressed in Equation (6)

(6) $$\left\{\begin{array}{c} Y_{j}^{R}=O^{R}W_{j}^{R}\\ Y_{j}^{G}=O^{G}W_{j}^{G}\\ Y_{j}^{B}=O^{B}W_{j}^{B} \end{array}\right.$$

where *j* represents the number of feature dimensions retained after dimensionality reduction.

In previous studies, researchers often selected the top Z(0<z<j) eigenvalues ranked from largest to smallest as the feature dimensions required for image reconstruction. However, in this paper, an improved BPSO algorithm will be utilized to intelligently select the projected feature vectors. After selecting the projected feature vectors, they are grouped into a set of projected feature vectors $B=\left[Y_{1},Y_{2},\ldots Y_{d}\right]$ and used in conjunction with the corresponding projection vectors $L=\left[W_{1},W_{2},\ldots W_{d}\right]$ to reconstruct the image. *d* denotes the number of projected feature vectors selected by the improved BPSO algorithm. The formula for image reconstruction is shown in Equation (7)

(7) $$\tilde{O}=BL^{T}=\sum_{k=1}^{d}\left(Y_{k}W_{k}^{T}\right)$$

$\bar{O}$ represents the reconstructed sub-image, indicating that the image *O* is approximately reconstructed through multiple sets of mutually exclusive projected feature vectors and projection vectors.

#### 3.2.2. Improved BPSO Algorithm

**Figure 2:**
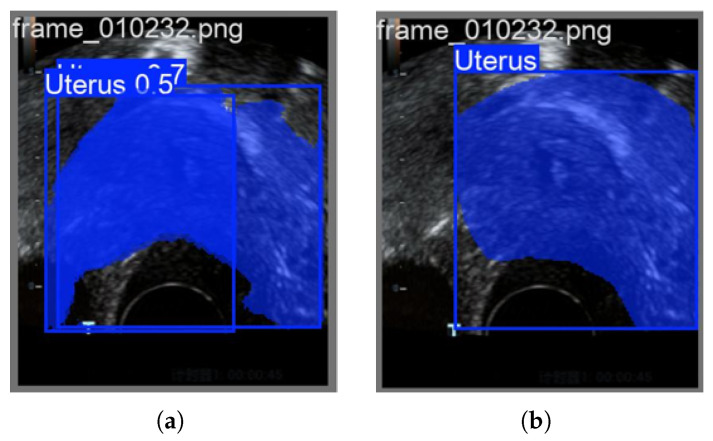
Prediction of traditional BPSO algorithm: (**a**) Traditional BPSO forecast. (**b**) Ground Truth.

As shown in Figure 2, the traditional BPSO algorithm often produces the phenomenon of redundant target frames when the features identified during feature selection process are applied to endometrial detection. This phenomenon differs from what is observed in practical applications, suggesting that the algorithm may not be able to distinguish valid features from interfering information. Therefore, in this work, the problems that traditional BPSO algorithms are prone to are improved as described below.

When selecting the projected feature vectors, the first step is to quantize them into a binary problem, transforming it from a continuous real-valued problem into a discrete space-constrained problem. Second, with the improved RGB-2DPCA algorithm, the dimensionality of the feature vectors has been extended to three channels, which requires adapting the algorithm to a version suitable for three channels.

In PSO, the positions of randomly initialized particles are generated as random floating-point numbers between 0 and 1. In BPSO, binary encodings are generated according to a specific strategy. The improved BPSO algorithm, tailored to the problem requirements and incorporating the principles of BPSO, initializes the positions of random particles as three-dimensional 0s or 1s. As for the initialization of the particles’ velocities, they are randomly set as three-dimensional values of −1, 0, or 1.

In the PSO algorithm, the update formulas for the particle’s position and velocity are shown in Equation (8) and Equation (9), respectively

(8) $$\begin{array}{c} v_{i}\left(t+1\right)=\omega \cdot v_{i}\left(t\right)+\alpha \cdot r_{1}\cdot \left(p_{{\mathrm{best}}_{i}}-x_{i}\left(t\right)\right)+\beta \cdot r_{2}\cdot \left(g_{\mathrm{best}\;}-x_{i}\left(t\right)\right) \end{array}$$

(9) $$x_{i}\left(t+1\right)=x_{i}\left(t\right)+v_{i}\left(t+1\right)$$

where $v_{i}\left(t\right)$ represents the velocity of the particle at the *t*-th iteration, and $x_{i}\left(t\right)$ represents the position of the particle at the *t*-th iteration. $p_{best_{i}}$ denotes the individual historical best position of the particle, while $g_{best}$ represents the global best position of the swarm. ω is the inertia weight, which controls the influence of the particle’s historical velocity. α and β are acceleration constants, governing individual cognition and social cognition, respectively. $r_{1}$ and $r_{2}$ are random numbers uniformly distributed between [0,1].

In BPSO, the velocity update formula is consistent with Equation (8). However, since the velocity $v_{i}\left(t\right)$ is a real number, it needs to be probabilistically mapped using the Sigmoid function, as shown in Equation (10)

(10) $$S\left(v_{i}\right)=\frac{1}{1+e^{-v_{i}}}$$

The calculated $S(v_{i})$ represents the probability of the particle’s next value. Thus, the particle position update formula for BPSO is shown in Equation (11)

(11) $$x_{i}\left(t+1\right)=\left\{\begin{array}{c} 1\;\;\mathrm{if}\;\mathrm{rand}\;\left(\right)<S\left(v_{i}\left(t+1\right)\right),\\ 0\;\;\mathrm{otherwise}\; \end{array}\right.$$

where rand() is a random number within the range of [0,1].

In the improved BPSO algorithm, the particle position update formula remains the same as Equation (9). For the velocity update formula, it is similar to Equation (8), but in the handling of $r_{1}$ and $r_{2}$, integers of 0 or 1 are directly generated randomly. After updating $v_{i}$, the sign function is used to clip its range, constraining the search space as shown in Equation (12).

(12) $$\mathrm{sgn}\left(v_{i}\right)=\left\{\begin{array}{cc} -1 & \;\mathrm{if}\;v_{i}<0\\ 0 & \;\mathrm{if}\;v_{i}=0\\ 1 & \;\mathrm{if}\;v_{i}>0 \end{array}\right.$$

In both PSO and BPSO, it is necessary to define a fitness function (objective function) to evaluate the solutions in the solution space and guide the optimization process. The improved BPSO is no exception. To this end, a fitness function incorporating reconstruction error and sparsity is defined, as shown in Equation (13), to select the projected feature vectors and projection vectors,

(13) $$\;\mathrm{Fitness}\;=∥X-\hat{X}{∥}_{F}+\lambda \cdot k$$

where *X* represents the original image, $\hat{X}$ denotes the reconstructed image, λ is the sparsity penalty coefficient used to control the sparsity weight in the selection of projected feature vectors and projection vectors, and *k* is the number of selected feature vectors. ${∥\cdot ∥}_{F}$ represents the Frobenius norm, defined as Equation (14):

(14) $${∥A∥}_{F}=\sqrt{\sum_{i=1}^{m}\sum_{j=1}^{n}{\left|a_{ij}\right|}^{2}}$$

where $A=\left[a_{ij}\right]$ is a matrix of size m×n. The Frobenius norm is used to measure the similarity or distortion between each channel of the image.

Using the improved BPSO algorithm, the projection feature vectors with the highest similarity across the three channels are identified to form $B_{1}$. Subsequently, the $\ell_{2}$ norm is computed for all projection feature vectors. The $\ell_{2}$ norm can be interpreted as energy or power, making it suitable for vector comparison. This is defined in Equation (15)

(15) $${∥X∥}_{2}=\sqrt{x_{1}^{2}+x_{2}^{2}+\ldots +x_{n}^{2}}$$

where the vector $X=\left[x_{1},x_{2},\ldots ,x_{n}\right]\in R^{n}$.

Finally, the top 100 largest vectors calculated by the $\ell_{2}$ norm are selected to form $B_{2}$. The union of $B_{1}$ and $B_{2}$ is taken to obtain the projected feature vectors *B* used for reconstructing the sub-images, as defined in formula Equation (16). The image is then reconstructed using formula Equation (7).

(16) $$B=B_{1}\cup B_{2}$$

### 3.3. Efficiency Analysis of BPSO Algorithm

We define the following notations:

- *N*: Number of particles;
- *T*: Maximum number of iterations;
- *d*: Feature dimension;
- $C_{r}$: Complexity of one image reconstruction process (e.g., RGB-2DPCA transformation);
- $C_{\ell_{2}}$: Complexity of computing the $\ell_{2}$ norm;
- $C_{s}$: Complexity of selecting the top 100 features (e.g., quicksort O(dlogd)).

Compared with the traditional BPSO algorithm, the improved BPSO introduces three-channel feature processing and additional vector selection mechanisms, which slightly increase the computational cost per iteration. However, by simplifying the velocity update using integer values and the sign function, the proposed method reduces the complexity of random sampling and probabilistic mapping. Furthermore, although $\ell_{2}$ norm computation and feature sorting introduce O(d) and O(dlogd) overhead respectively, they are only applied to selected subsets and have limited impact on the overall efficiency. Therefore, as shown in Table 1 the improved algorithm maintains a comparable order of time complexity while enhancing optimization convergence and feature discrimination capability.

## 4. Experiments

### 4.1. Dataset

The experiment utilized normal endometrial imaging data obtained through transvaginal contrast-enhanced ultrasound provided by the Department of Ultrasound at Changshu Hospital Affiliated to Nantong University. The dataset comprises 2770 endometrial images (1385 conventional sonographic images and 1385 contrast-enhanced images) obtained from 14 patients and segmented at one-second intervals. These images exhibit significant heterogeneity in parameters such as endometrial proportion, positional orientation, dimensional measurements, and background characteristics.

Following the conventional method of dataset partitioning, the image data from the 14 patients were randomly divided into training, validation, and test subsets at ratios of 70%, 20%, and 10%, respectively. These subsets were then aggregated to form the final training set, validation set, and test set required for the experiment.

### 4.2. Introduction to Platform and Parameter Settings

The same evaluation metrics as the YOLO model were adopted, namely Box mAP and Mask mAP, recorded as mAP50-95 for both Box and Mask. Through experimentation, it was found that mAP50 was less accurate in reflecting evaluation results when the dataset size was small, and thus it was not used.

All experiments employed the same hyperparameters. For YOLO, the SGD optimizer was chosen, with an initial learning rate (lr0) of 0.01, 20 epochs, and a random seed set to 2025. In the improved BPSO algorithm, the swarm size was set to 50, with 100 iterations, ω set to 1.5, α to 2, and β to 1. The selection of these parameters is informed by prior research [22,23,24,25] and guided by empirical insights specific to this problem domain.

In Equation (13), the value of λ is set to 0.001. Considering that the Frobenius norm in the fitness function has already been normalized, the value of λ was initially compared among 0, 0.001, 0.01, 0.1, and 1. As shown in Table 2, experimental results indicate that variations within the range of 0–0.001 lead to positive performance changes. Therefore, the optimal parameter value was sought within this range; however, due to time constraints, only odd-numbered values were tested. As shown in Table 3, all evaluation experiments were conducted using k-fold cross-validation (with k = 10 in this case) to assess the generalization performance of the data.

In the comparative experiments, following the traditional 2DPCA approach, the top 160 vectors corresponding to the largest eigenvalues were selected to form the projected feature vectors for the experiment.

### 4.3. Comparative Experiment

A total of 31 comparative experiments were conducted. For ease of description, the different models in the experiments are named as follows:

- The conventional ultrasound model without any processing is denoted as Original-N.
- The contrast-enhanced ultrasound (CEUS) model without any processing is denoted as Original-CEUS.
- The grayscale fusion images of conventional sonographic and contrast-enhanced ultrasound, reconstructed using the top 160 eigenvectors from traditional 2DPCA, are named based on fusion ratios: TradFusion-x, where x ranges from 0.1 to 0.9, representing the proportion of contrast-enhanced ultrasound images increasing from 10% to 90%, while the proportion of conventional ultrasound images decreases correspondingly, maintaining a complementary relationship.
- Similarly, the color fusion images of conventional and contrast-enhanced ultrasound, reconstructed by applying 2DPCA separately to RGB channels using the top 160 eigenvectors, are designated as RGBTradFusion-x.
- For the fusion images reconstructed using vectors selected by the improved BPSO algorithm, the naming follows the same rule and is denoted as FLFSI-Fusion-x.
- For images using the YOLOv12 model, the naming follows the same rule and is recorded as YOLOv12-FLFSI-Fusion-x.

The experimental data analysis based on the Table 4 demonstrates that the effective integration of SI and feature-level image fusion methods enhances model performance. Using Original-N (93.8% Box mAP/85.9% Mask mAP) as the benchmark, the TradFusion traditional grayscale image 2DPCA series achieved approximately a 0.8% accuracy improvement in Mask mAP and a 2.3% increase in Box mAP through feature fusion. This result indicates that feature-level fusion of conventional and contrast-enhanced ultrasound (CEUS) images can strengthen the feature representation capability of endometrial imaging.

However, it is noteworthy that as the fusion threshold parameter increases (0.1 → 0.9), model performance exhibits a declining trend: Mask mAP drops continuously from 86.7% to 71.6% (a 15.1% decline), while Box mAP decreases by 5.5%. Similar trends are observed in the RGBTradFusion and FLFSI-Fusion series. This performance degradation is hypothesized to result from the absence of blood flow features in early contrast agent perfusion stages, where ultrasound instruments fail to receive feedback signals, thereby weakening regional detection accuracy.

The comparative analysis between RGBTradFusion and the TradFusion series shows that the integration of RGB channels into the traditional 2DPCA frame increases the accuracy of both Box mAP and Mask mAP. In addition, RGBTradFusion shows less performance degradation in both Box mAP and Mask mAP metrics under varying threshold conditions. This comparison confirms that the color channel feature fusion approach has stronger threshold invariance and better cross-threshold generalizability compared to grayscale-based 2DPCA. In particular, in complex medical imaging scenarios requiring multi-threshold analysis, the color information fusion mechanism significantly improves threshold-robust feature representations by leveraging multispectral correlations in color space to mitigate performance variations caused by parameter fluctuations.

A comparison between the FLFSI-Fusion and RGBTradFusion series demonstrates that the improved BPSO algorithm, which selects identical projection feature vectors across three channels and performs feature completion, achieves more outstanding accuracy. Specifically, at x=0.2, FLFSI-Fusion achieves peak performance in both Mask mAP (87.8%) and Box mAP (96.6%). Training image analysis suggests that fusing 20% weighted CEUS images enhances structural discernibility of endometrial regions by effectively complementing specific frequency-band echo signals with tissue texture features.

Furthermore, integrating the FLFSI-Fusion feature-level image fusion strategy into the YOLOv12 detection framework further elevates the model’s overall performance. As shown in Table 4, *YOLOv12-FLFSI-Fusion-0.2* achieves the highest scores across all methods, with a Box mAP of 96.6% and a Mask mAP of 88.8%.

### 4.4. Generalization Verification Experiment

Cross-validation (CV) is a common approach for determining the optimal number of components in a principal component analysis model [26,27,28,29]. Using 10-fold cross-validation, the proposed FLFSI method was evaluated for generalization performance on both YOLOv11 and YOLOv12. As shown in Table 5, the FLFSI method achieved consistently strong detection results across both detection models, demonstrating its favorable generalization capability. Specifically, YOLO11-FLFSI-Fusion-0.1 attained an average Box mAP of 98.37% and an average Mask mAP of 93.76%, while YOLO11-FLFSI-Fusion-0.2 delivered 98.02% and 93.66%, respectively; the YOLOv12 counterparts-YOLOv12-FLFSI-Fusion-0.1 and YOLOv12-FLFSI-Fusion-0.2-reached 98.24% and 92.62% and 98.17% and 92.73% for Box and Mask mAP, both outperforming the baseline Original-N (95.21% and 86.72%) and Original-CEUS (91.73% and 74.55%) by clear margins. Moreover, the low standard deviations (Box SD ≤ 0.0073, Mask SD ≤ 0.0125) across all FLFSI variants indicate stable performance across folds.

The same rule was also applied to TradFusion and RGBTradFusion for completeness. *TradFusion-0.1* achieved 97.41% Box mAP and 87.95% Mask mAP with the lowest Box SD (0.0069) among non-FLFSI methods, confirming its reliability; *TradFusion-0.2* yielded 96.12% and 87.11% while exhibiting even tighter variance (Box SD 0.0055, Mask SD 0.0068). By introducing the RGB channels, *RGBTradFusion-0.1* climbed to 97.68% Box mAP and 88.73% Mask mAP, and *RGBTradFusion-0.2* reached 97.85% and 87.67%. Although their standard deviations are marginally higher (Box SD up to 0.0095, Mask SD up to 0.0176), both RGB-enhanced models consistently surpassed the baselines and maintained cross-fold stability, evidencing that the additional color cues improve accuracy without sacrificing robustness.

### 4.5. Comparative Analysis of Predictive Performance in Experiments

**Figure 3:**
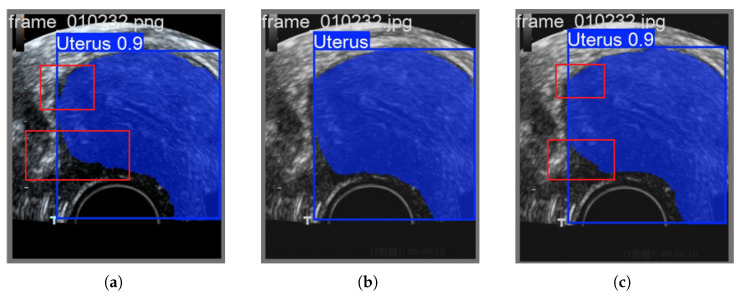
Advantages of the feature fusion image of traditional 2DPCA compared to the baseline: (**a**) Original-N Forecast. (**b**) Ground Truth. (**c**) TradFusion-0.1 Forecast.

The YOLO11 baseline model suffers from inaccurate region detection. As indicated by the red box in the Figure 3, the feature-level image fusion method demonstrated in the TradFusion series can effectively address these inaccuracies in region detection.

**Figure 4:**
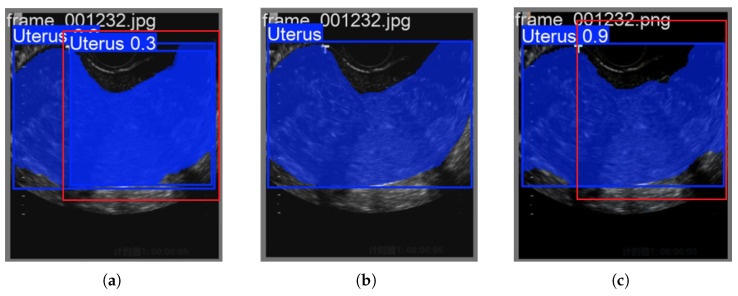
The traditional 2DPCA method suffers from the problem of excessive organ prediction: (**a**) TradFusion-0.1 Forecast. (**b**) Ground Truth. (**c**) RGBTradFusion-0.1 Forecast.

Through the Figure 4, it is observed that the images reconstructed by the traditional 2DPCA-based fusion method tend to exhibit the aforementioned issue of over-detection in some cases. However, this phenomenon has not been noted in the RGBTradFusion series, which fully utilizes color space information.

**Figure 5:**
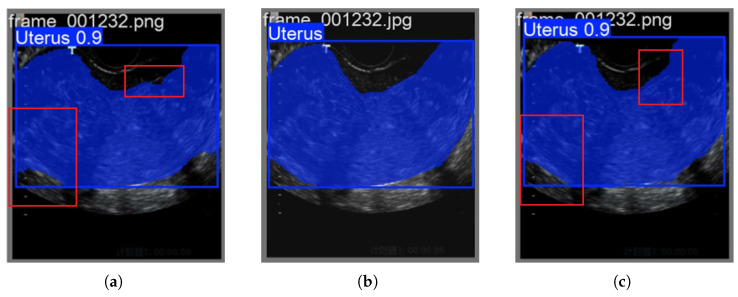
Advantages of FLFSI: (**a**) TradFusion-0.1 Forecast. (**b**) Ground Truth. (**c**) FLFSI-Fusion-0.1 Forecast.

As shown in Figure 5, it is noteworthy that although the RGBTradFusion series has achieved improvements in region detection accuracy compared to the traditional 2DPCA method and baseline models, there remains a slight performance gap when contrasted with the FLFSI-Fusion series. This further demonstrates that the FLFSI method not only enhances the feature representation capability of endometrial tissues in imaging but also improves the robustness of the model.

**Figure 6:**
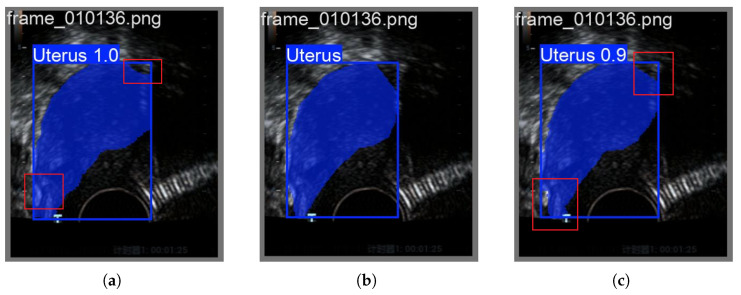
Advantages of YOLOv12-FLFSI: (**a**) FLFSI-Fusion-0.2 Forecast. (**b**) Ground Truth. (**c**) YOLOv12-FLFSI-Fusion-0.2 Forecast.

As shown in Figure 6, by applying our FLFSI method to the YOLOv12 model, we observed a further improvement in the detection and segmentation of the endometrial region compared to its performance with the YOLO11 model. The introduction of attention mechanisms enables more refined feature extraction in key regions, enhancing the delineation of endometrial boundaries.

### 4.6. Feature Selection Time Comparison Between Traditional BPSO Algorithm and Improved BPSO Algorithm

As discussed in Section 3.3, although the traditional BPSO algorithm and the improved BPSO algorithm are similar in theoretical time complexity, our experimental results reveal that the improved BPSO algorithm exhibits a clear advantage in actual running time. Specifically, we measured the time consumed during feature selection for both algorithms. The results, shown in Table 6, demonstrate that the improved BPSO algorithm achieves better efficiency in practice.

### 4.7. Analysis of Detection Effects of Traditional BPSO and Improved BPSO After Feature Selection

The proposed improved BPSO surpasses the traditional variant in endometrial-region detection by virtue of its accurate discrete feature-selection modeling and efficient optimization. The conventional BPSO employs a sigmoid-based probabilistic update that maps velocities into (0, 1), causing position updates to rely heavily on probability and rendering the algorithm prone to local optima-particularly in the 640-dimensional sparse feature space. This limitation is reflected in Table 7, where *YOLO11-FLFSI-Fusion-0.1* achieves only 81.93% Box mAP and 60.39% Mask mAP. In contrast, the improved BPSO introduces a sign function to enable discrete jump updates, directly quantizing velocities into integer steps −1, 0, 1. Coupled with tri-channel binary particle encoding and zero-initialized velocity, this strategy strengthens global exploration. Consequently, the proposed method attains 98.37% Box mAP and 93.76% Mask mAP in 10-fold cross-validation (Table 5), representing an improvement of over 16 percentage points, while standard deviations decrease to 0.0042 (Box) and 0.0094 (Mask). These results firmly validate the algorithm’s superior convergence stability and feature-selection accuracy.

Moreover, traditional BPSO-based feature selection could potentially be augmented with dynamic attention-augmented transformer architectures, as demonstrated in gaze-driven human–machine collaboration systems for complex manufacturing environments [30]. Such architectures incorporate temporal modeling and adaptive attention mechanisms to process variable-quality and noisy input streams in real time. If integrated into the conventional BPSO framework, this approach could improve robustness against acquisition noise and signal variability—issues often present in clinical CEUS imaging of the endometrial region. However, even with such enhancements, the probabilistic position updates of standard BPSO may still hinder global exploration in high-dimensional sparse spaces, underscoring the necessity of the proposed discrete velocity-quantization strategy for achieving stable convergence and high-precision feature selection.

## 5. Conclusions

The new method proposed in this paper, FLFSI, fully exploits the multidimensional information of conventional sonography and contrast-enhanced sonography within the color space. By optimizing the selection of projection vectors, it improves the accuracy of endometrial region detection. In practice, conventional sonography is limited by factors such as the wavelength and energy of the ultrasound. This often leads to blurring at the edges and interference due to reflections from the surrounding organs, which limits the localization of lesions. In contrast, contrast-enhanced imaging techniques can enhance the differences in blood flow between lesions and surrounding tissue, providing clearer image information.Experimental results validate the effectiveness of this method through feature-level fusion in color space and swarm intelligence optimization algorithms, while clinical datasets demonstrate its potential and practical value in improving the detection of endometrial regions.

At present, the utilisation of dynamic imaging time-series features is still insufficient, which is mainly manifested by the fact that current methods can only process images frame by frame, failing to fully explore the time-series information. In addition, the selection of existing intelligent evolutionary algorithms is relatively simple, and their feature screening is limited to a single dataset, resulting in insufficient generalisation capability, which may require more complex optimisation strategies when expanding to a wider range of datasets. Future research will focus on the fusion of temporal model-based and deep learning algorithms to process video streams of the whole process of contrast agent infusion, as well as exploring new applications of intelligent evolutionary algorithms in medical image analysis. In addition, Generative Adversarial Network (GAN)-based low-quality image enhancement modules will be constructed to improve the robustness of the models to optimise the performance and adaptability of medical image analysis. 

## Figures and Tables

**Table 1:** Time Complexity Comparison Between Traditional BPSO and Improved BPSO.

Step	Traditional BPSO	Improved BPSO.
Initialization	O(N·d)	O(N·d·3)
Velocity update	O(N·d) (float + sigmoid)	O(N·d) (integer random + sign function)
Position update	O(N·d) (sigmoid + sampling)	O(N·d) (simplified sign-based update)
Fitness evaluation	$O(N\cdot C_{r})$	$O(N\cdot \left(C_{r}+C_{\ell_{2}}+C_{s}\right))$
Overall per iteration	$O(N\cdot \left(d+C_{r}\right))$	$O(N\cdot \left(d+C_{r}+C_{\ell_{2}}+C_{s}\right))$
**Total complexity**	$O(N\cdot T\cdot \left(d+C_{r}\right))$	$O(N\cdot T\cdot \left(d+C_{r}+C_{\ell_{2}}+C_{s}\right))$

**Table 2:** Results at Different Orders of Magnitude.

Magnitude	Avg. Box mAP	Avg. Mask mAP
0	97.14%	93.05%
**0.001**	**98.25**%	**94.02**%
0.01	96.18%	92.54%
0.1	91.42%	88.25%
1	85.73%	80.18%

**Table 3:** Results in the Range 0.0003–0.001.

Magnitude	Avg. Box mAP	Avg. Mask mAP
0.0003	97.12%	93.02%
0.0005	97.48%	93.28%
0.0007	97.86%	93.65%
0.0009	98.05%	93.88%
**0.001**	**98.25**%	**94.02**%

**Table 4:** Comparative Experiment Table.

Methods	Box mAP	Mask mAP
Original-N	93.8%	85.9%
Original-CEUS	90.5%	73.9%
TradFusion-0.1	96.1%	86.7%
TradFusion-0.2	94.9%	86.3%
TradFusion-0.3	93.9%	85.5%
TradFusion-0.4	93.4%	83.6%
TradFusion-0.5	92.4%	82.7%
TradFusion-0.6	93.8%	77.6%
TradFusion-0.7	91.5%	74.5%
TradFusion-0.8	90.4%	72.4%
TradFusion-0.9	90.6%	71.6%
RGBTradFusion-0.1	**96.1**%	**87.5**%
RGBTradFusion-0.2	**96.5**%	**86.8**%
RGBTradFusion-0.3	94.9%	85.6%
RGBTradFusion-0.4	94.0%	85.1%
RGBTradFusion-0.5	94.6%	83.5%
RGBTradFusion-0.6	92.3%	81.4%
RGBTradFusion-0.7	92.1%	78.8%
RGBTradFusion-0.8	90.8%	76.0%
RGBTradFusion-0.9	90.9%	73.6%
FLFSI-Fusion-0.1	**96.2**%	**87.7**%
FLFSI-Fusion-0.2	**96.6**%	**87.8**%
FLFSI-Fusion-0.3	93.5%	85.4%
FLFSI-Fusion-0.4	94.6%	85.2%
FLFSI-Fusion-0.5	93.3%	82.3%
FLFSI-Fusion-0.6	91.2%	81.2%
FLFSI-Fusion-0.7	92.7%	80.1%
FLFSI-Fusion-0.8	90.7%	75.0%
FLFSI-Fusion-0.9	89.8%	73.7%
YOLOv12-FLFSI-Fusion-0.1	95.2%	86.9%
YOLOv12-FLFSI-Fusion-0.2	**96.6**%	**88.8**%

**Table 5:** 10-fold cross-validation results of the above key methods.

Methods	Avg. Box mAP	Avg. Mask mAP	SD (Box)	SD (Mask)
Original-N	95.21%	86.72%	0.0086	0.0121
Original-CEUS	91.73%	74.55%	0.0212	0.0315
TradFusion-0.1	97.41%	87.95%	0.0069	0.0088
TradFusion-0.2	96.12%	87.11%	0.0055	0.0068
RGBTradFusion-0.1	97.68%	88.73%	0.0095	0.0176
RGBTradFusion-0.2	97.85%	87.67%	0.0088	0.0119
YOLO11-FLFSI-Fusion-0.1	98.37%	93.76%	0.0042	0.0094
YOLO11-FLFSI-Fusion-0.2	98.02%	93.66%	0.0072	0.0125
YOLOv12-FLFSI-Fusion-0.1	98.24%	92.62%	0.0054	0.0114
YOLOv12-FLFSI-Fusion-0.2	98.17%	92.73%	0.0073	0.0125

**Table 6:** Comparative Time Table.

Time	Traditional BPSO	Improved BPSO
CEUS	2152.11 min	**540.9** min
Conventional	1338.65 min	**435.72** min

**Table 7:** Detection effect table of traditional BPSO and improved BPSO after feature selection.

Methods	Avg. Box mAP	Avg. Mask mAP	SD (Box)	SD (Mask)
YOLO11-FLFSI-Fusion-0.1	81.93%	60.39%	0.0219	0.0231
YOLO11-FLFSI-Fusion-0.2	80.68%	59.18%	0.0378	0.0285
YOLOv12-FLFSI-Fusion-0.1	77.55%	54.41%	0.0316	0.0450
YOLOv12-FLFSI-Fusion-0.2	78.07%	53.81%	0.0362	0.0376

## Data Availability

The original contributions presented in this study are included in the article. Further inquiries can be directed to the corresponding author.
